# Trace Levels of Innate Immune Response Modulating Impurities (IIRMIs) Synergize to Break Tolerance to Therapeutic Proteins

**DOI:** 10.1371/journal.pone.0015252

**Published:** 2010-12-22

**Authors:** Daniela Verthelyi, Vivian Wang

**Affiliations:** Division of Therapeutic Proteins, Office of Biotechnology Products, Center for Drug Evaluation and Research, Food and Drug Administration, Bethesda, Maryland, United States of America; Johns Hopkins School of Medicine, United States of America

## Abstract

Therapeutic proteins such as monoclonal antibodies, replacement enzymes and toxins have significantly improved the therapeutic options for multiple diseases, including cancer and inflammatory diseases as well as enzyme deficiencies and inborn errors of metabolism. However, immune responses to these products are frequent and can seriously impact their safety and efficacy. Of the many factors that can impact protein immunogenicity, this study focuses on the role of innate immune response modulating impurities (IIRMIs) that could be present despite product purification and whether these impurities can synergize to facilitate an immunogenic response to therapeutic proteins. Using lipopolysaccharide (LPS) and CpG ODN as IIRMIs we showed that trace levels of these impurities synergized to induce IgM, IFNγ, TNFα and IL-6 expression. *In vivo*, trace levels of these impurities synergized to increase antigen-specific IgG antibodies to ovalbumin. Further, whereas mice treated with human erythropoietin showed a transient increase in hematocrit, those that received human erythropoietin containing low levels of IIRMIs had reduced response to erythropoietin after the 1^st^ dose and developed long-lasting anemia following subsequent doses. This suggests that the presence of IIRMIs facilitated a breach in tolerance to the endogenous mouse erythropoietin. Overall, these studies indicate that the risk of enhancing immunogenicity should be considered when establishing acceptance limits of IIRMIs for therapeutic proteins.

## Introduction

Biotechnology products, in many cases recombinant proteins, are derived from complex expression/production systems that often involve genetically modified host cells (bacteria, yeast, insect or mammalian) and complex growth/fermentation media [1]. The ensuing purification steps may be insufficient to completely eliminate impurities such as DNA, host cell proteins, or endotoxins from the product. As a result, biologics may contain low levels of host cell or process derived impurities [2]. These impurities, which we designate as Innate Immune Response Modifying Impurities (IIRMIs), can potentially stimulate the innate immune system via a host of pattern recognition receptors and foster the development of an immune response to the product. Immune responses to protein therapeutic products, including the production of anti-drug antibodies or cell-based immune responses, are frequent [3], [4]. While some immune responses do not appear to have clinical implications, others result in infusion reactions (e.g. hypersensitivity), alter the drug's pharmacokinetics, neutralize or otherwise compromise the clinical safety or efficacy of a product [1], [5]. Such is the case for patients who receive replacement therapies for hemophilia or inborn errors of metabolism such as Pompe's disease, where the development of neutralizing antibodies to the replacement therapy deprives the patients from a life saving therapy [6]–[8]. In addition, immune responses to therapeutic proteins can result in loss of tolerance to their endogenous counterparts leading to serious adverse events [1]. Examples of these include the development of pure red cell aplasia in patients treated with Eprex® [9], and of thrombocytopenia in patients that received as few as 2 doses of a truncated and pegylated recombinant thrombopoietin [10].

Immune cells express numerous receptors that respond to conserved microbial molecular patterns [11]–[13]. The family of Toll like receptors (TLR) recognizes distinct highly conserved microbial components. For example, TLR 4 recognizes lipopolysaccharide (LPS), TLR 9 is activated by DNA encoding unmethylated cytosine-phosphate guanine motifs (CpG motifs), and TLR 3 responds to double-stranded RNA [14]. Multiple studies show that ligands for these TLR can be used to increase the immunogenicity of foreign proteins [15] and may facilitate the breaking of tolerance to self [16]. Indeed, data from the field of vaccine adjuvants shows that in mice, addition of 25–100 µg of CpG ODN or 10–100 µg of LPS to a protein antigen potentiates the production of antigen-specific antibodies, T cell survival and Th1 differentiation to the protein [17]–[20]. Several studies have further shown that low levels of peptidoglycans or TLR 7 agonists can synergize with TLR 3 or TLR 4 agonists to foster secretion of IL-12 and other cytokines by dendritic cells (DC) [21]–[25] and increase the immunogenicity of vaccines [26]. However, to date, there are no formal studies addressing the impact of combinations of very low level TLR agonists on the immunogenicity of therapeutic proteins.

In this study we hypothesized that TLR-stimulating impurities, at levels close to the limit of detection by currently used analytical methods, may stimulate the immune system and impact the product in terms of its immunogenicity, particularly when more than one type of impurity is present. Using LPS and synthetic DNA strands containing CpG motifs as a model we determined that trace levels of TLR 4 and 9 agonists synergize to induce cellular activation with increased cytokine and chemokine expression and antibody production both *in vitro* and *in vivo*.

## Materials and Methods

### Reagents and media

Phosphorothioate CpG ODN (GCTAGACGTTAGCGT) and Control ODN (GCTAGAGCTTAGGCT) were synthesized at the CBER core facility. All ODN had less than <0.1 EU of LPS per mg of ODN as assessed by a Limulus amebocyte lysate assay (QCL-1000, BioWhittaker, Walkersville, MD). *E. coli* LPS (0111:B4) were purchased from InvivoGen (San Diego, CA) and used per manufacturer's instructions. The stated potency of the endotoxin is 1EU/ng. Ultrapure Ovalbumin (Ovalbumin grade V, OVA) was obtained from Sigma-Aldrich. Ovalbumin used to immunize mice was a kind gift from Brian Kelsall (NIAID, NIH) and contained less than 0.025EU of endotoxin per 5 µg OVA as assessed by the LAL assay. Human Erythropoietin (rhuEPO) was purchased from GenScript Corporation (Piscataway, NJ). Endotoxin content in rhuEPO was measured using LAL Assay as above.

### Mice and experimental design

Animal studies were conducted under protocol 2006-43 as approved by the White Oak Animal care and Use Committee of the FDA. Mice used for study were obtained from the National Cancer Institute (Frederick, MD). In one study, 2 month old female BALB/c mice (n = 5/group) were immunized with 5 µg of OVA alone or together with different amounts of CpG ODN or LPS as specified for each experiment. The animals were boosted 3 weeks later with the same preparation. Mice that received saline were used as controls. Mice were tail-bled before the immunization and weekly thereafter. For another study, two month old female C57BL/6 mice (n = 5/group) received 5 µg of rhuEPO alone or together with the TLR agonists (i.p.). Mice that received saline served as controls to establish the natural variability of the hematocrit and the impact of serial tail bleeding. After first dose, blood was collected and HCT was measured weekly/biweekly. Animals were re-treated 14 and 62 days after first injection. Percent of increased/reduced hematocrit was calculated relative to the baseline for each individual mouse.

### Quantitative Real-Time PCR (q-RT-PCR)

Total RNA was prepared from splenocytes using TRIzol (Invitrogen, Paisley, Scotland, UK) as per manufacturer instructions and then purified with RNAeasy (Qiagen, Valencia, CA). Subsequently, RNA (500 ng/sample) was reverse transcribed into cDNA using a High Capacity cDNA Reverse Transcription Kit (Applied Biosystems Inc., Foster City, CA, USA) as per manufacturer's instructions. cDNA samples were treated RNase H (Invitrogen) for 30 minutes at 37°C and stored at −20°C until used for q-RT-PCR. Expression values were calculated using the 2^−ΔΔ*Ct*^ method [27]. For the mRNA array, total RNA was then reverse transcribed and subsequently analyzed on TaqMan Low Density Array cards by TaqMan PCR using a 7900HT (ABI) as per manufacturer's instruction.

### Cytokine and Antibody Assays

Supernatants: 5×10^5^ splenocytes/well were cultured in RPMI 10% FCS media at 37°C for 48 hr. IFNγ, IL-6, and Ig were assessed by using 96-well plates (Immunolon, Thermo LabSystems, Franklin MA) coated with cytokine-specific antibody or antigen and then blocked with PBS-1% bovine serum albumin (Sigma, St Louis, MO). After washing, the plates were overlaid with the supernatant for 3 hours, then further washed, and treated with the appropriate biotinylated secondary antibody, followed by AKP-conjugated avidin (BD, Biosciences, Franklin Lakes, NJ). Absorbance was read at 405nm.

### Statistical analysis

Changes in antibody or cytokine expression were analyzed by t test, ANOVA or repeated measure ANOVA as appropriate. Differences in hematocrit were evaluated by SAS using a mixed model with repeated measures where HCT = treat+day+treat*day+treat*day^2^+treat*day^3^. P values<0.05 were considered significant.

## Results

### Substimulatory levels of LPS and CpG ODN synergize to induce an immune response

**Figure 1:**
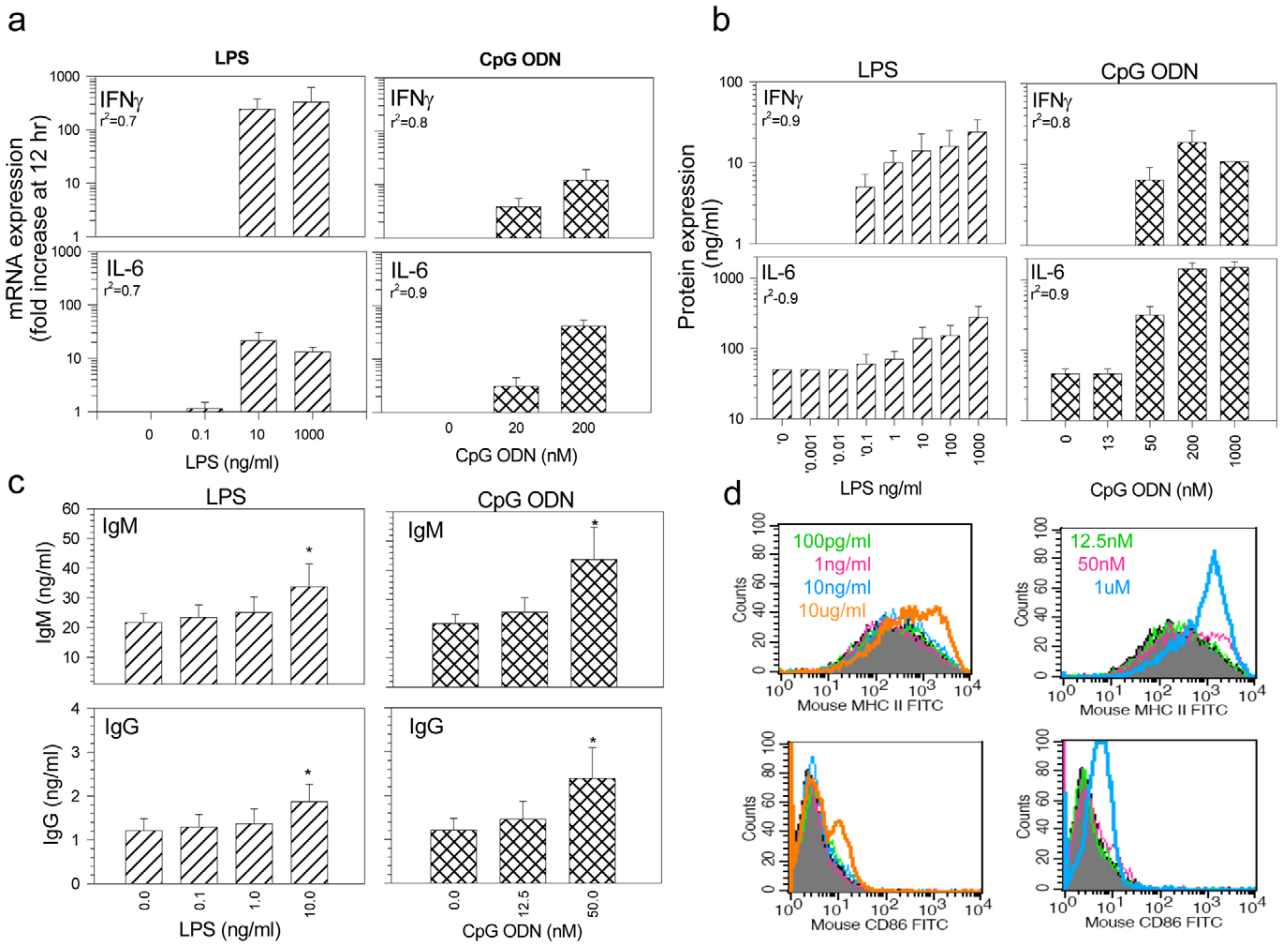
Induction of IL-6 and IFNγ by increasing concentrations of LPS and CpG ODN. Balb/c splenocytes (2×10^6^/ml; n = 2–5) were placed in culture with increasing concentrations of LPS or CpG ODN. a. Fold increase in mRNA relative to unstimulated cells from the same animal were determined by q-RT-PCR after 12 hours. b/c. IFNγ and IL-6 and total immunoglobulin levels in 48 hr supernatants as determined by ELISA. d. shows the expression of MHC-II and CD86 in CD19^+^-CD220^+^ B cells 48 hours after stimulation in vitro. Shown is 1 of 3 experiments with similar results. The dose dependent increase in mRNA and protein expression was tested by repeated measures ANOVA and regression analysis.

Endotoxin and host cell DNA are impurities routinely monitored in the manufacture of biologics. Both can stimulate the innate immune system via the TLR system and function as adjuvants enhancing the immunogenicity of proteins by activating antigen presenting cells, inducing cytokine secretion and directing polyclonal B cell activation [11]. *In vitro*, optimal stimulation of splenocytes can be achieved using 10 µg/ml LPS or 1–3 µM (4–12 µg/ml) CpG ODN. However, as shown in figure 1, concentrations of LPS as low as 10 ng/ml (10 EU/ml) consistently stimulated increased mRNA and protein expression for IL-6 and IFNγ in Balb/c splenocytes, while 20–50 nM (0.02–0.05µg/ml) of synthetic DNA encoding a CpG motif were sufficient to induce these cytokines *in vitro*. Splenocytes from C57Bl/6 mice exhibited similar results (*data not shown*). These trace levels of TLR 4 and 9 agonists also elicited B cells activation as evidenced by an increase in total IgM and IgG output and upregulation of MHC class II and CD86 expression (fig 1c&d).

**Figure 2:**
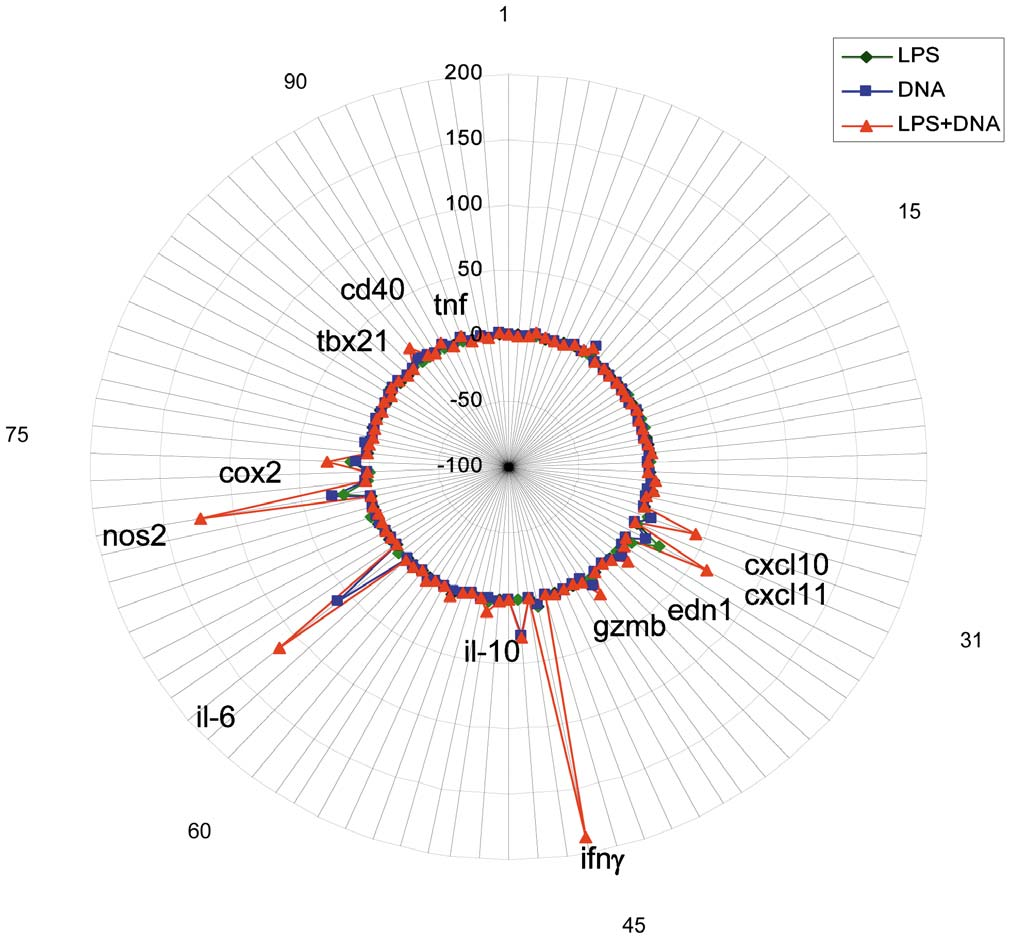
Suboptimal levels of LPS and CpG ODN synergize to induce the expression of IFN-related and proinflammatory genes in splenocytes. Balb/c Splenocytes were placed in culture in the presence of low levels of LPS (10ng/ml) and/or CpG ODN (20nM). The breadth of mRNA expression was determined using TaqMan low density DNA arrays (TLDA). A complete list of genes is available in supplemental Table S1. Genes identified in the graph showed a significant increase in gene expression (p<0.05) and a mean fold increase >5 relative to unstimulated cells. Differences in gene expression were assessed by ANOVA.

**Figure 3:**
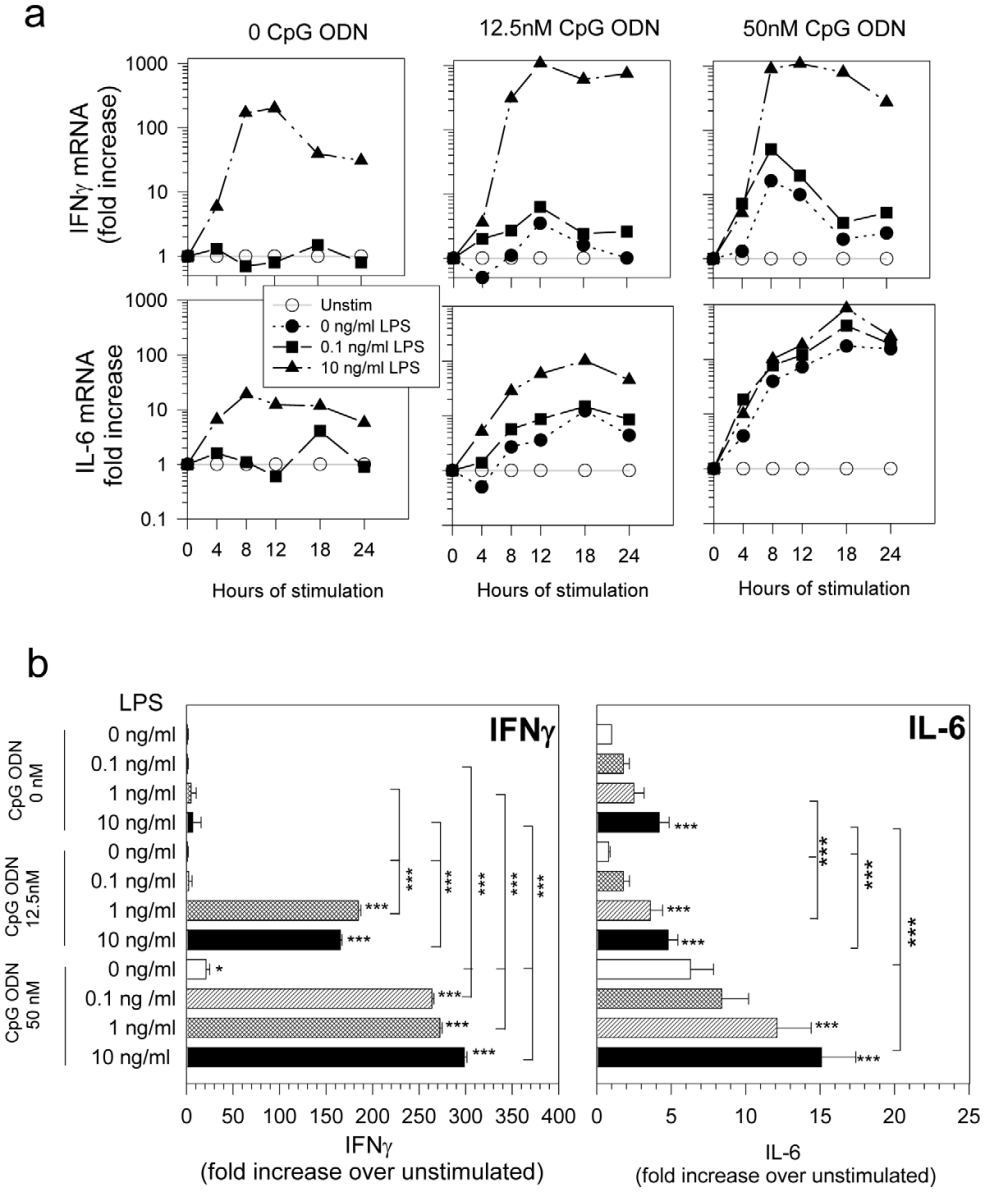
IFNγ and IL-6 expression in cells stimulated with trace levels of LPS and CpG ODN. Splenocytes were cultured in the presence of increasing concentrations of LPS and CpG ODN alone or in combination. a. The expression of mRNA was monitored for 24 hr and is shown as fold-increase over time 0. b. Protein expression in culture supernatants was determined at 48 hr. LPS concentrations are expressed in ng/ml. Statistical significance (* p<0.05; ***p<0.001): differences between treatment groups are noted as horizontal stars signal significant increase relative to the corresponding concentration of CpG ODN, or as vertical stars, which show significant increase of dual stimulation over the corresponding concentration of each individual stimulant (CpG ODN or LPS) (ANOVA, p<0.001).

To assess whether the presence of low levels of both IIRMIs together would further increase immune activation, splenocytes were stimulated with the lowest levels of LPS (10ng/ml) and CpG ODN (20 nM) that induced a reproducible response *in vitro*. The breadth of the activation was determined using TaqMan low density DNA arrays (TLDA) for 92 immune-related genes (supplementary Table S1). As shown in figure 2, the addition of both stimuli together triggered a significant increase in mRNA for TBx21, IFNγ and IFNγ- inducible proteins, CXCL10 and CXCL11, as well as interleukin 6 (IL-6), granzyme B, nitric oxide synthase 2 (nos2) and cycloxygenase 2 (cox2) suggesting that the presence of these IIRMIs fosters a pro-inflammatory/type I response. In contrast, while individually LPS and CpG ODN induced small increases in IL-4, IL-5 and IL-10 mRNA expression, no synergy was evident. The increased mRNA expression was sustained for at least 24 hours and resulted in dose-dependent increases in IFNγ and IL-6 protein concentration in culture supernatants, as shown in figure 3a. In the presence of sub-stimulatory levels of CpG ODN (12.5 nM), concentrations of LPS (as low as 1ng/ml or 0.1EU) significantly increased IFNγ and IL-6 production.

#### Submitogenic levels of LPS and CpG ODN synergize to enhance antibody responses to proteins

**Figure 4:**
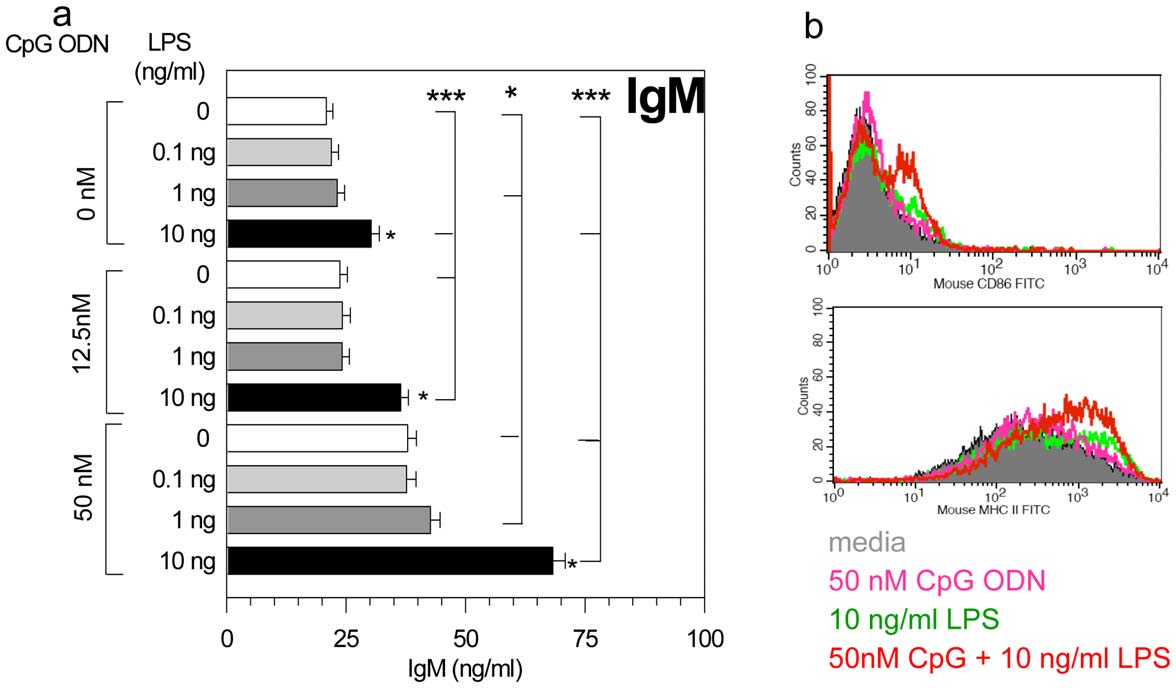
B cells activation by trace levels of LPS and CpG ODN. Balb/c splenocytes were cultured in the presence of increasing concentrations of LPS and CpG ODN alone or in combination. a. Total IgM expression in culture supernatants was determined at 48 h by ELISA. LPS concentrations are expressed in ng/ml. Statistical analysis: Repeated measure ANOVA*p<0.05, ***p<0.001.b. Cell surface expression of MHC-II and CD86 on CD220^+^ B cells. Show is 1 of 3 experiments with similar results.

In B cells, TLR activation results in the up-regulation of activation markers, proliferation, cytokine secretion, terminal differentiation into plasma cells and, finally, immunoglobulin secretion [28]–[32]. When used as adjuvants, TLR agonists were shown to augment B cell responses to specific protein antigens even in the absence of capable T cell help [33]. To establish whether the presence of substimulatory levels of LPS and CpG DNA would promote polyclonal B cell activation, splenocytes were stimulated with low levels of LPS and CpG DNA as described above. As shown in figure 4, TLR agonists synergized to increase the secretion of total IgM (figure 4a) and the expression of CD86, CD69 and MHC II on B cells (figure 4b).

#### Protein immunogenicity *in vivo*

**Figure 5:**
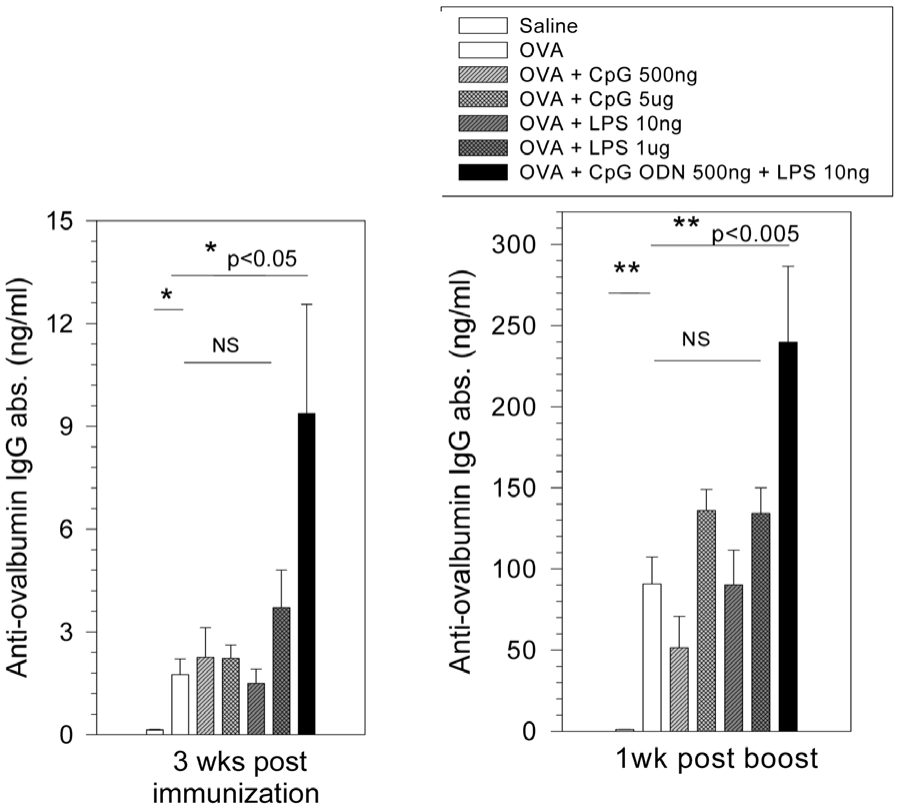
Trace levels of IIRMIs increase protein immunogencity *in vivo*. Balb/c mice (3–6 week old; 5 mice/group) were immunized and boosted i.p. with human ovalbumin alone (5µg/mouse) or mixed with the stated amounts of LPS and/or CpG ODN. Mice that received saline were used as controls. Mice were tail-bled before the immunization and weekly thereafter. Antibodies (IgG) to ovalbumin assessed by ELISA. Statistical analysis: ANOVA. * = p<0.05, ** p<0.005, NS: Not significant.

Numerous studies have demonstrated that TLR 4 and TLR 9 agonists in optimal amounts (10–100 µg for LPS and 25–100 µg for CpG ODN) significantly increased the efficacy of vaccines in murine models [17], [34]–[36]. To determine whether trace amounts of TLR agonists were sufficient to increase the immunogenicity of a foreign protein in rodents, Balb/c mice were immunized with LPS-free ovalbumin (5 µg/mouse, subcutaneously) alone or together with LPS (10 ng–1µg/animal) or CpG ODN (500 ng–5µg/animal) individually, or the combination. As shown in figure 5, administration of ovalbumin alone or together with trace or suboptimal amounts of a single TLR-agonist induced very low levels of IgG antibodies. In contrast, mice that received the same dose of ovalbumin together with 10 ng of LPS plus 500 ng of CpG ODN had significantly increased (p<0.05) IgG antibody responses to ovalbumin 3 weeks post treatment. The same group showed significantly higher antibody titers following re-exposure 4 weeks after priming (p<0.005). Indeed, the antibody levels in these mice were significantly higher than those immunized with ovalbumin together with either 5 µg of CpG ODN or 1 µg of LPS (p<0.05).

#### TLR-binding impurities can synergize to break tolerance to endogenous proteins

**Figure 6:**
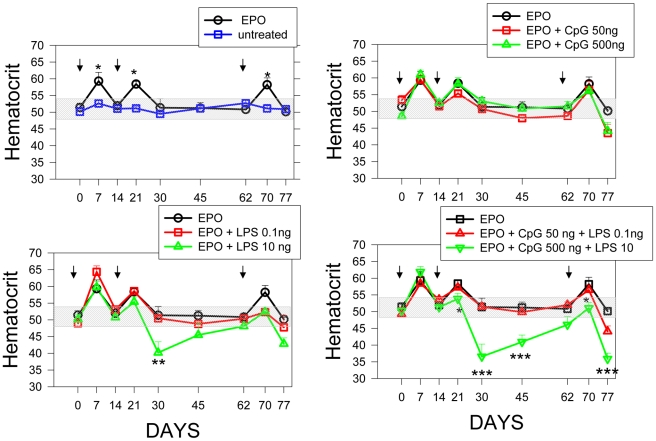
Impurities can synergize to break tolerance to “self” proteins. Two month-old female C57BL/6 mice (n = 5/group) received rhuEPO (5 µg i.p. on day 0, 14 and 62) alone or together CpG ODN and/or LPS as shown (black arrows), and then bled (50 µl) every 7 or 14 days. Mice that received saline served as controls. Results are expressed as the mean (±SD) hematocrit for each individual mouse (Mean ± SEM). The gray box shows the mean +3SD of the hematocrit of untreated mice over the time of the experiment. Those mice were bled with the same schedule as the treated groups. Statistical analysis: SAS mixed model with repeated measures where HCT = treat+day+treat*day+treat*day^2^+treat*day^3^. P values<0.05 were considered significant. * p<0.05; *** p<0.01.

Tolerance to low-abundance self proteins in sera is incomplete and may be overcome when these proteins are presented in the context of adequate adjuvants [37]–[39]. Using a model established by Ryan et al. [40], we determined whether the addition of low levels of LPS and/or CpG DNA was sufficient to induce a breach in tolerance and the induction of a neutralizing response to erythropoietin. Balb/c mice were treated with recombinant Human Erythropoietin (rhuEPO) alone or together with low levels of LPS and/or CpG ODN on days 0, 14 and 62, followed by weekly hematocrit measurements. As shown in figure 6, the hematocrit of untreated mice remains constant over time (51±3%). Despite having only 80% homology with mouse [41], rhuEPO was active in mice, eliciting a reproducible increase in the hematocrit 1 week after each treatment (15.5, 13.9 and 13.6% increase over baseline after the 1^st^, 2^nd^ and 3^rd^ dose respectively). Addition of low levels of CpG ODN (50 or 500 ng), LPS (100 pg) or the combination (50 ng CpG ODN +100pg LPS) did not modify the response. In mice treated with rhuEPO together with LPS (10ng) there was evidence of reduced hematocrit following the 2^nd^ inoculation. In contrast, mice treated with rhuEPO in combination with 500 ng of CpG ODN plus 10 ng of LPS showed reduced response to rhuEPO following one treatment as evidenced by increases in hematocrit of 23, 7 and 1.5% over baseline after the 1^st^, 2^nd^ and 3^rd^ inoculations respectively, followed by a pronounced reduction in hematocrit (2%, 27% and 28% respectively relative to baseline; p<0.001). The reduction in the hematocrit lasted for over 30 days following the 2^nd^ inoculation. This data shows that the rhuEPO augments the hematocrit of mice for about 7 days. The prolonged anemia observed in mice treated with rhuEPO plus LPS and CpG ODN suggests that the inoculations led to a break in tolerance to the endogenous (mouse) EPO, which is needed to maintain the hematocrit stable. Together these data suggests that in mice, the presence of low levels of impurities that can trigger TLR 4 and 9 are may foster a break in tolerance to an essential endogenous non-redundant growth factor.

## Discussion

In the vaccine field, the concept of immunostimulatory combinations is being actively explored and studies using combinations of TLR and NLR agonists have shown promise in efforts to overcome tolerance to tumor antigens [42]. In therapeutic proteins, where immune responses are not desired, factors that can contribute to product immunogenicity such as the degree of pre-existing tolerance, post-translation modifications, glycosylation patterns, and the presence of aggregates are carefully monitored [3]. The data above shows that process-related impurities, especially those of microbial origin, may also play a key role in the immunogenicity of therapeutic proteins. The effect of IIRMIs was dose dependent and synergistic as levels of CpG ODN and LPS that individually induced no or very low levels of cytokine release by murine splenocytes, elicited polyclonal B cell activation with increased antigen-specific immunoglobulin and pro-inflammatory/Th1 cytokine output as well as up-regulation of co-stimulatory molecules on the cell surface of antigen presenting cells (Figure 4 & data not shown). This synergistic effect was then confirmed *in vivo*, as studies showed that the combination of 10 ng of LPS and 500 ng of CpG ODN, which do not induce an immune response when present individually, were sufficient to promote the immunogenicity of proteins and contribute to a clinically relevant break in tolerance to self.

Of note, this study was not designed to establish the minimal concentration of TLR -binding impurities that could render a protein immunogenic in humans, but to explore the potential for increased protein immunogenicity when trace levels of multiple impurities are present in a product. The minimal amount of TLR 4 and 9 agonists that can synergize to act as adjuvants in mice can not be used to calculate the minimal level of impurities that can increase protein immunogenicity in Man as i. animal models do not predict immunogenicity in humans [1]; ii. mice and humans differ significantly in their TLR distribution and sensitivity to agonists [43]; and iii, humans are likely to be much more sensitive to TLR agonists than rodents [44]. This is shown in studies using CpG ODN as vaccine adjuvants, where 25–100 µg of CpG ODN are used in mice weighing ∼25–30 g compared to 100 µg–1mg of CpG ODN in adult humans.

During the manufacture of therapeutic proteins a few IIRMIs, such as LPS and DNA, are regularly screened-for and have assigned limits or acceptance criteria for product release. Of note, the current guidelines for setting limits on these impurities are not based on their potential impact on product immunogenicity. For example, the current recommendation for endotoxin content in parenteral products (0.5 EU/kg/hr) is based on its pyrogenic potential, while the WHO recommendations for DNA content (<10 ng DNA/dose) are based on minimizing the risk of DNA integration (USP <85> and [45]). Yet studies using individual TLR 4 or TLR 9 agonists as adjuvants show that concentrations lower than those that may be pyrogenic or lead to significant DNA integration can augment the immune response to co-administered protein antigens [17], [19], [28]. Furthermore, as shown in the studies above, the levels of agonists sufficient to stimulate an innate response can be lower when multiple receptors are engaged.

Previous studies had shown that combinations of TLR and/or NLR agonists in vitro increased the production of pro-inflammatory cytokines such as IL-6, TNFα, IL-12 and type I IFNs by macrophages and DC [21], [46]–[48]. DC activated in this fashion had prolonged expression of IκBξ, sustained upregulation of costimulatory molecules leading to the activation of polarized antigen-specific CD4+ T cells, and reduced the regulatory effects of CD4^+^ CD25^+^ Tregs on CD8^+^ T cell responses *in vitro* [21], [48], [49].

The precise mechanisms by which low levels of endotoxin and DNA synergize to foster the activation of anergic B and T cells specific for low-abundance self-antigens is still unknown at this time. The synergy could result from enhanced activation and cooperation among NF-κB, IRF, MAPK, PI-3K, and STAT signaling pathways. TLR 9 is known to use Myeloid differentiation primary response gene (88) (MyD88) while LPS can use both MyD88 and TIR-domain-containing adapter-inducing interferon-β (TRIF) as adaptor molecules. Of note, previous studies cautioned that multiple stimuli that signal via the same downstream pathway (such as MyD88 ) could result in a dampened response to TLR agonists [50]. This led to hypothesize a “combinatorial code” whereby TLR agonists that signaled via MyD88 could synergize with those that used a different transduction pathway such as TRIF [21]. Similar predictions were made for IIRMs that signaled via Interferon regulatory factor 3 (IRF3) or IRF7 [21], [51]. However, if the dampening effect of multiple innate immune agonists is mediated by competition for adaptor molecules, this restriction may not apply to trace level impurities and therefore synergy may be evident regardless of shared signaling paths. In addition, the enhancement could be indirect as our preliminary studies show that TLR agonists can upregulate other TLR receptors increasing the sensitivity to low levels of ligand.

It is well established that the generation of immune responses to therapeutic proteins can affect both the safety and efficacy of life-saving products. Neutralizing antibody responses to therapeutic proteins are particularly problematic when the endogenous counterpart of the therapeutic protein serves a biologically unique function. For instance, erythropoietin is a non-redundant growth factor that stimulates and controls the development of erythroid progenitors. It is present in human sera in very low levels (12–15mU/ml) [52]. Between 1999 and 2002, following a manufacturing change, 206 patients treated with erythropoietin developed pure red cell aplasia (PRCA) [53]. The increase in product immunogenicity was attributed to a possible increase in leachables from the container closure, but the precise factors that combined to increase the immunogenicity in a small fraction of the patients receiving treatment are still unresolved [54]. In our model, the presence of very low levels of CpG ODN and LPS in rhuEPO was sufficient to induce a clinically relevant reduction in hematocrit. Previous studies have shown that administration of high levels of CpG ODN (100 µg/mouse) can lead to a short term reduction in hematocrit that was mediated by an increase in IFNγ [55]. Our study differs from Thawani et al's in that the levels of CpG ODN administered are 200 fold lower and do not induce *per se* a reduction in the hematocrit. Further, in our model, the reduction in hematocrit was more severe following a second or third administration strongly suggesting that the reduction in hematocrit was immune –and not inflammatory- mediated. Of note, the pro-hematopietic effect of the rhuEPO in our mice lasted for about 1 week. In contrast, the hematocrit reduction following the administration of rhuEPO plus LPS and CpG ODN lasted for 4–6 weeks. This suggests that anemia is the result of an immune response that neutralized with activity of both the rhuEPO and the mouse erythropoietin for an extended period. Together these data suggest that the presence of trace levels of IIRMIs may facilitate an immune response to a foreign protein and foster a break in tolerance to a relatively homologous endogenous protein which may cause a severe, albeit transient, deficiency syndrome.

In summary, our data suggests that levels of impurities previously not known to contribute to product immunogenicity may synergize to foster the response to a therapeutic protein. These findings underscore the need to establish sensitive assays to quantify the levels of any impurities that could activate the innate immune system.

## Supporting Information

Table S1 Trace levels of LPS and CpG ODN synergize to induce mRNA expression of selected genes.
(DOC)
