# Supplementary material for: Trace Levels of Innate Immune Response Modulating Impurities (IIRMIs) Synergize to Break Tolerance to Therapeutic Proteins
Source: PLoS One. 2010 Dec 22;5(12):e15252. doi: 10.1371/journal.pone.0015252 (PMC3008684; doi:10.1371/journal.pone.0015252)
Supplement: Table S1 — Trace levels of LPS and CpG ODN synergize to induce mRNA expression of selected genes. (DOC) [file pone.0015252.s001.doc]

| GENE | LPS | CpG ODN | LPS+CpG ODN |  | GENE | LPS | | CpG ODN | | LPS+CpG ODN | |  | |
| --- | --- | --- | --- | --- | --- | --- | --- | --- | --- | --- | --- | --- | --- |
| B2m | 1.2 | 1.3 | 1.6 |  | Ifng | 8.3 | | 7.3 | | 132.0 | | ** | |
| Bax | 1.1 | 2.7 | 3.3 |  | Ikbkb | 0.7 | | 0.7 | | 0.7 | |  | |
| Bcl2 | 0.7 | 1.4 | 1.5 |  | Il10 | 1.5 | | 27.3 | | 27.1 | |  | |
| Bcl2l1 | 0.9 | 0.9 | 1.0 |  | Il12a | 1.0 | | 1.1 | | 1.2 | |  | |
| C3 | 2.2 | 1.2 | 1.2 |  | Il12b | 0.6 | | 1.8 | | 1.7 | |  | |
| Ccl19 | 2.5 | 3.6 | 1.8 |  | Il13 | 13.5 | | 5.1 | | 15.7 | |  | |
| Ccl2 | 1.9 | 2.3 | 2.9 |  | Il15 | 1.2 | | 0.8 | | 1.0 | |  | |
| Ccl3 | 2.8 | 8.4 | 6.7 |  | Il17 | 0.4 | | 0.0 | | 0.0 | |  | |
| Ccl5 | 1.2 | 1.0 | 1.2 |  | Il18 | 0.9 | | 0.7 | | 0.7 | |  | |
| Ccr2 | 1.3 | 1.0 | 1.2 |  | Il1a | 5.1 | | 2.1 | | 4.3 | |  | |
| Ccr4 | 1.1 | 0.7 | 0.6 |  | Il1b | 3.0 | | 1.9 | | 2.0 | |  | |
| Ccr7 | 1.0 | 1.3 | 1.5 |  | Il2 | 1.3 | | 0.6 | | 0.6 | |  | |
| Cd19 | 1.1 | 0.9 | 0.8 |  | Il2ra | 1.2 | | 2.2 | | 4.0 | |  | |
| Cd28 | 0.6 | 0.3 | 0.3 |  | Il3 | 0.1 | | 0.2 | | 0.1 | |  | |
| Cd34 | 1.2 | 0.4 | 0.6 |  | Il4 | 2.7 | | 2.2 | | 3.2 | |  | |
| Cd38 | 1.1 | 1.2 | 1.2 |  | Il5 | 8.6 | | 0.3 | | 9.5 | |  | |
| Cd3e | 1.0 | 0.5 | 0.6 |  | Il6 | 2.5 | | 54.2 | | 88.4 | | ** | |
| Cd4 | 1.1 | 0.6 | 0.5 |  | Il7 | 0.3 | | 0.5 | | 0.1 | |  | |
| Cd40 | 1.6 | 3.9 | 4.5 |  | Lrp2 | 2.6 | | 1.9 | | 0.5 | |  | |
| Cd40lg | 0.6 | 0.3 | 0.2 |  | Lta | 2.0 | | 1.0 | | 0.0 | |  | |
| Cd68 | 0.7 | 0.5 | 0.4 |  | Nfkb1 | 0.9 | | 1.4 | | 1.8 | |  | |
| Cd80 | 1.4 | 0.8 | 1.0 |  | Nfkb2 | 1.3 | | 1.7 | | 1.5 | |  | |
| Cd86 | 1.3 | 0.9 | 1.7 |  | Nos2 | 19.2 | | 25.2 | | 102.5 | | ** | |
| Cd8a | 0.9 | 0.5 | 0.6 |  | Pgk1 | 1.3 | | 2.5 | | 2.9 | |  | |
| Col4a5 | 1.0 | 0.3 | 0.1 |  | Prf1 | 0.7 | | 0.7 | | 1.0 | |  | |
| Csf1 | 2.7 | 2.1 | 4.0 |  | Ptgs2 | 10.3 | | 8.1 | | 20.9 | |  | |
| Csf2 | 2.8 | 1.0 | 3.7 |  | Ptprc | 1.0 | | 0.6 | | 0.8 | |  | |
| Csf3 | 0.6 | 0.5 | 1.3 |  | Sele | 1.4 | | 0.8 | | 1.0 | |  | |
| Ctla4 | 1.1 | 0.9 | 1.1 |  | Selp | 0.8 | | 0.2 | | 0.4 | |  | |
| Cxcl10 | 5.2 | 8.6 | 34.1 | ** | Ski | 0.7 | | 0.4 | | 0.5 | |  | |
| Cxcl11 | 84.4 | 44.3 | 209.4 | ** | Smad3 | 0.9 | | 0.7 | | 0.8 | |  | |
| Cxcr3 | 1.0 | 0.5 | 0.5 |  | Smad7 | 0.7 | | 0.4 | | 0.3 | |  | |
| Cyp1a2 | 23.7 | 0.0 | 0.0 |  | Socs1 | 1.8 | | 1.5 | | 3.4 | |  | |
| Cyp7a1 | 0.6 | 1.6 | 0.0 |  | Socs2 | 1.1 | | 0.7 | | 0.6 | |  | |
| Ece1 | 1.2 | 1.7 | 1.9 |  | Stat1 | 3.9 | | 2.8 | | 3.2 | |  | |
| Edn1 | 1.1 | 7.2 | 12.2 | * | Stat3 | 1.3 | | 2.0 | | 2.0 | |  | |
| Fas | 1.5 | 1.6 | 1.9 |  | Stat4 | 1.0 | | 0.5 | | 0.5 | |  | |
| Fasl | 1.0 | 0.7 | 0.9 |  | Stat6 | 0.2 | | 0.0 | | 1.3 | |  | |
| Fn1 | 0.3 | 0.2 | 0.1 |  | Tbx21 | 1.6 | | 4.6 | | 14.5 | | ** | |
| Gusb | 0.9 | 1.4 | 1.2 |  | Tfrc | 1.0 | | 2.2 | | 1.9 | |  | |
| Gzmb | 2.0 | 7.0 | 12.3 | * | Tgfb1 | 0.9 | | 0.8 | | 0.7 | |  | |
| H2-Ea | 0.5 | 10.7 | 0.3 |  | Tnf | 1.8 | | 5.3 | | 6.4 | |  | |
| H2-Eb1 | 1.2 | 0.6 | 0.5 |  | Tnfrsf18 | 1.0 | | 0.6 | | 0.6 | |  | |
| Hmox1 | 0.9 | 1.3 | 0.9 |  | Vcam1 | 0.5 | | 0.3 | | 0.2 | |  | |
| Hprt1 | 1.2 | 2.7 | 2.8 |  | Vegfa | | 1.1 | | 1.8 | | 0.2 | |  |
| Icos | 1.2 | 0.8 | 0.7 |  | Gapdh | | 1.2 | | 1.5 | | 1 | |  |

Trace levels of LPS and CpG ODN synergize to induce mRNA expression of selected genes. Splenocytes (n=3 independent experiments) were cultured for 12 h in the presence of LPS (10ng/ml)and/or CpG ODN (20nM). levels of RNA expression were determined using a TaqMan Low Density Array cards by TaqMan PCR using a 7900HT. Statistical significance was established by ANOVA * p<0.05, ** p<0.01.
